# Safety and Efficacy of Atropine 0.05% Versus 0.01% for Prevention of Myopic Progression in Indian Children: A Randomized Clinical Trial

**DOI:** 10.7759/cureus.84010

**Published:** 2025-05-13

**Authors:** Siddharam S Janti, Kalpana Mali, Eereti Sahithi, Antarvedi Tejaswini, Bhushan Kamble, Srividya Kalluri

**Affiliations:** 1 Ophthalmology, All India Institute of Medical Sciences, Bibinagar, Bibinagar, IND; 2 Pharmacology, Neelima Institute of Medical Sciences, Ghatkesar, IND; 3 Community and Family Medicine, All India Institute of Medical Sciences, Bibinagar, Bibinagar, IND

**Keywords:** accommodation, atropine 0.01%, axial length, photophobia, progressive myopia

## Abstract

Aim

This study was conducted to evaluate the effectiveness and safety of 0.01% and 0.05% atropine eye drops in managing myopia progression in children compared to a placebo.

Methods

This randomized, interventional study was conducted from March 2022 to May 2023 at the All India Institute of Medical Sciences, Bibinagar, Telangana. A total of 272 children aged five to 16 years, with myopia ranging from -1.0 D to -6.0 D and an annual progression greater than 0.5 D, were enrolled. Participants were randomly assigned to three groups: Group A (n=88, atropine 0.01%), Group B (n=90, atropine 0.05%), and Group C (n=94, placebo). Comprehensive ophthalmic examinations, including cycloplegic refraction, axial length measurement, and fundus evaluation, were performed at baseline, six weeks, 12 weeks, and at the end of one year. Changes in refractive error, axial length, accommodation, and pupil size were analyzed.

Results

Both atropine treatments were effective in slowing myopia progression compared to the placebo. Atropine 0.05% significantly reduced refractive progression (0.263 ± 0.03 D) and axial elongation (0.138 ± 0.22 mm) compared to the placebo group (0.759 ± 0.8 D and 0.367 ± 0.33 mm, respectively). The 0.01% atropine group also demonstrated a reduction, although less pronounced (0.319 ± 0.05 D and 0.241 ± 0.22 mm, respectively). Both atropine groups exhibited an increase in the near point of accommodation and pupil size, with the 0.05% group showing more significant changes. Mild adverse effects, including near vision difficulties and photophobia, were reported but did not significantly impact patient compliance.

Conclusion

Atropine 0.05% is more effective than 0.01% atropine and placebo in slowing myopia progression in children. Although both concentrations led to increased near point of accommodation and pupil size, side effects were mild and well-tolerated.

## Introduction

Myopia is a common refractive error characterized by the focusing of light in front of the retina rather than directly on it. It can be corrected with spectacles, contact lenses, or refractive surgery. However, monitoring the progression of myopia is critical, as uncontrolled progression can lead to severe complications such as choroidal neovascularization and retinal detachment [1]. Myopia typically begins in childhood and tends to stabilize during the teenage years, but early intervention is crucial to prevent or slow its progression.

In India, myopia has emerged as a significant public health concern among school-aged adolescents in both urban and rural settings, warranting urgent attention. The increasing prevalence of myopia is potentially associated with the rising use of digital devices such as smartphones and tablets [2], although this relationship requires further investigation. Evidence suggests that excessive near work is linked to an increased risk of developing myopia [3].

Over the past four decades, the crude prevalence of myopia among children aged five to 15 years in India has been approximately 7.5%, with a higher prevalence noted in urban areas (8.5%) compared to rural areas (6.1%). The highest rates are observed among urban children aged 11-15 years [4]. Several strategies are available to manage the progression of myopia, including rigid gas permeable contact lenses and bifocal lenses. However, pharmacological intervention with atropine has been identified as the most effective method to slow myopia progression [5].

## Materials and methods

This randomized, interventional case study was conducted from 2022 to 2023 at the All India Institute of Medical Sciences (AIIMS), Bibinagar, Telangana, after obtaining approval from the Institutional Ethics Committee, AIIMS, Bibinagar (Approval No. AIIMS/IEC/SEP/2021/72) and registration with the Clinical Trials Registry of India (CTRI/2021/11/038177). The study aimed to evaluate the efficacy of different concentrations of atropine eye drops in managing childhood myopia.

The sample size was calculated using G*Power software (version 3.1.9.4) (Heinrich-Heine-Universität Düsseldorf, Düsseldorf, Germany) based on the expected effect size (Cohen’s d) of 0.5, alpha (α) of 0.05, and power (1-β) of 80%. Considering a 10% dropout rate, the required total sample size was determined to be approximately 270 participants, with at least 90 participants per group.

A total of 272 children aged five to 16 years with confirmed myopia ranging from -1.0 D to -6.0 D and an annual progression exceeding 0.5 D were enrolled. Exclusion criteria included astigmatism greater than 1.5 D, amblyopia, prior intraocular surgery, known allergy to atropine, systemic conditions associated with myopia (e.g., Marfan syndrome, Stickler syndrome), significant cardiac or respiratory diseases, and unwillingness to provide informed consent.

After initial spectacle correction, participants were randomized into three groups using a computer-generated random number sequence with block randomization. Allocation concealment was ensured through sequentially numbered, sealed, opaque envelopes. Outcome assessments were conducted by a co-investigator blinded to treatment allocation. Cycloplegic refraction was performed using 1% cyclopentolate eye drops.

Participants were assigned to three groups. Group A (n=88) received atropine 0.01%, Group B (n=90) received atropine 0.05%, and Group C (n=94) received placebo drops containing carboxymethyl cellulose (CMC) 0.5%.

Randomization and recruitment were conducted through the ophthalmology outpatient department at AIIMS Bibinagar. Comprehensive ophthalmic evaluations were performed at baseline, six weeks, 12 weeks, and at one year, including cycloplegic refraction (auto-refractometer, keratometer, and retinoscopy), intraocular pressure measurement (non-contact tonometry), fundus examination (using a Volk® +90 diopter lens with slit-lamp biomicroscopy; Volk Optical Inc., Mentor, OH, USA and indirect ophthalmoscopy), anterior chamber depth, pupillary diameter, axial length (measured using Zeiss IOL Master 700, Carl Zeiss Meditec AG, Jena, Germany), and amplitude of accommodation (measured using a Royal Air Force (RAF) ruler). Participants in all three groups were instructed to administer the assigned eyedrops (atropine 0.01% in Group A, atropine 0.05% in Group B, and placebo in Group C) once daily at bedtime, continuously for one year.

At the end of the study, efficacy was assessed based on myopia progression, axial elongation, pharmacological side effects, and drug compliance. Additionally, participants and guardians completed a questionnaire regarding visual symptoms, including reading difficulty, diplopia, light sensitivity, and glare.

Statistical analysis was performed using IBM SPSS Statistics for Windows, Version 28 (IBM Corp., Armonk, NY). Descriptive statistics included mean±standard deviation (Mean±SD) for continuous variables and frequencies (percentages) for categorical variables. Inferential statistics involved the chi-square test (χ²) for categorical variables, one-way ANOVA for comparing continuous variables among the three groups at baseline and follow-up visits, followed by post-hoc Tukey’s HSD (Honestly Significant Difference) tests for pairwise comparisons. A p-value <0.05 was considered statistically significant.

## Results

The study enrolled 272 children between March 2022 and May 2023, with 88 participants in Group A (0.01% atropine), 90 in Group B (0.05% atropine), and 94 in Group C (placebo). Baseline characteristics, including age, sex, refractive error, axial length, near point of accommodation (NPA), and pupil size, were comparable across the three groups (Table 1).

**Table 1:** Demographic characteristics and optical quality of the study population before treatment. NPA: near point of accommodation. Statistical tests: Chi-square test (categorical variables) and one-way ANOVA (continuous variables).

Characteristics	Group A (n=88)	Group B (n=90)	Group C (n=94)	Test statistic	P-value
Sex					
Female	46	43	48	χ² = 0.32	0.85
Male	42	47	46		
Age in years (Mean (±SD))	8.88 (2.22)	9.0 (2.18)	8.50 (2.05)	F = 0.78	0.457
Pre-refraction in diopters (Mean (±SD))	2.38 (0.65)	2.30 (0.87)	2.14 (0.92)	F = 0.02	0.982
Pre-axial length in mm (Mean (±SD))	23.0 (1.11)	22.9(0.94)	22.1(0.90)	F = 1.20	0.305
NPA in cm (Mean (±SD))	6.50 (0.42)	6.56 (046)	6.70 (0.51)	F = 1.38	0.255
Pupil size in mm (Mean (±SD))	3.54 (0.25)	3.30 (0.41)	3.60 (0.19)	F = 2.89	0.057

Both atropine groups demonstrated significantly reduced myopic progression and axial elongation compared to the placebo group. Group B (0.05% atropine) showed the greatest reduction (Table 2).

**Table 2:** Comparison of changes in refractive error, axial length, accommodation, and pupil size among groups at one year after treatment. * One-way ANOVA test.

Variables	Group A (n=88) Mean (±SD)	Group B (n=90) Mean (±SD)	Group C (n=94) Mean (±SD)	F-value	P-value*
Rate of refractive growth in diopters	0.319 (0.05)	0.263 (0.03)	0.759 (0.8)	28.45	<0.01
Axial elongation in mm	0.241 (0.22)	0.138 (0.22)	0.367 (0.33)	14.22	<0.01

Group B had the highest percentage of children with the slowest progression (<0.25 D/year), while Group C had the highest proportion with the fastest progression (≥1 D/year) (Table 3).

**Table 3:** Distribution of myopia progressive rates between three groups. D: diopters; Y: year.

Myopia progression rates as per change of D/Y	Group A (n=88) No (%)	Group B (n=90) No (%)	Group C (n=94) No (%)
Slowest (<0.25)	22 (25)	39 (43.3)	13 (13.9)
Moderate (0.25-0.5)	12 (13.6)	20 (22.2)	14 (14.9)
Faster (0.50-1)	21 (23.9)	18 (20)	27 (28.7)
Fastest (≥1)	33 (37.5)	13 (14.4)	40 (42.5)

Atropine use led to a significant increase in NPA and pupil size, more prominently in Group B. Photophobia and near vision difficulties were slightly more common in the atropine groups but remained infrequent (Table 4).

**Table 4:** Comparison of treatment-related side effects among groups at one year after treatment. NPA: Near point of accommodation. * One-way ANOVA test. Statistical tests: Chi-square test (categorical variables) and one-way ANOVA (continuous variables).

Variables	Group A (n=88) Mean (±SD)	Group B (n=90) Mean (±SD)	Group C (n=94) Mean (±SD)	F-value	P-value*
Accommodation					
NPA before atropine use in cm (Mean (±SD))	6.50 (0.42)	6.56 (0.46)	6.70 (0.51)	1.38	0.255
NPA after atropine use (cm) (Mean (±SD))	7.20 (0.22)	7.80 (0.34)	6.70 (0.51)	9.58	<0.01
NPA change in %	10	18	0	19.45	
Near vision difficulty in %	1	3	0	χ²=2.78	
Pupil size					
Pupil size before atropine use in cm (Mean (±SD))	3.54 (0.25)	3.30 (0.41)	3.60 (0.19)	2.89	0.057

## Discussion

Myopia, apart from causing diminution of vision and significantly impacting quality of life, can lead to serious ocular complications. The risk of these complications is elevated not only in cases of high myopia but also in low to moderate myopia. Myopic patients have been shown to have a 100-fold higher risk of developing myopic macular degeneration (MMD), a threefold higher risk of retinal detachment (RD) and posterior subcapsular cataract (PSC), and an almost twofold increased risk of open-angle glaucoma (OAG). Other complications such as retinoschisis, macular holes, and staphylomas may occur if myopia remains untreated [6].

Preventing myopia progression is a major area of focus in ophthalmology and optometry. Several interventions have been explored to manage and slow myopia progression, particularly in children. The available options include orthokeratology, multifocal and bifocal contact lenses, atropine eye drops, increased time outdoors, and myopia control glasses [7-11].

Among these, atropine eye drops have emerged as a widely studied pharmacological intervention. Although the exact mechanism of action of atropine is not fully understood, it is believed to act via nonaccommodative pathways involving the nicotinic pathway, affecting biochemical processes within the retina and sclera. It may inhibit glycosaminoglycan synthesis in scleral fibroblasts and affect scleral remodeling. Additionally, atropine-induced mydriasis might increase ultraviolet light exposure to the peripheral retina, potentially limiting axial elongation. Some evidence also suggests that atropine may reduce chronic ocular inflammation associated with myopia progression [12].

While most previous studies have compared 0.01% atropine with placebo and demonstrated its benefit over no treatment, limited data are available regarding the effects of 0.05% atropine. Our study is the first randomized study comparing 0.01%, 0.05% atropine, and placebo specifically in an Indian pediatric population.

Effects of atropine on myopia progression

In our study, the mean myopia progression with 0.01% atropine was 0.319 ± 0.05 D, which is comparable to findings by Yam et al. (-0.43 D) [13], Chia et al. (1.38±0.98 D) [9], Clark and Clark (0.1 D/year) [14], Moon and Shin (-0.84 D/year) [15], and Wei et al. (−0.49±0.42 D) [16]. Similarly, Zadnik et al. found a change of −1.04 D in the 0.01% atropine group compared to −1.28 D in the placebo group at 36 months [17].

For the 0.05% atropine group, our study demonstrated a mean progression of 0.263 ± 0.03 D, closely matching the 0.28 D reported by Yam et al. and the -0.23 D reported by Moon and Shin [13,15].

In the placebo group, the mean progression was higher at 0.759 ± 0.8 D, which is comparable to Yam et al.'s findings of -0.76 D in untreated patients [13].

Effects of atropine on axial length elongation

Regarding axial length, we found mean elongations of: (i) 0.138 ± 0.22 mm in the 0.05% atropine group; (ii) 0.241 ± 0.22 mm in the 0.01% atropine group; and (iii) 0.367 ± 0.33 mm in the placebo group

These results are consistent with findings by Yam et al., who reported larger axial length changes in the placebo group (0.41±0.22 mm) compared to the 0.05% (0.20±0.25 mm), 0.025% (0.29±0.20 mm), and 0.01% (0.36±0.29 mm) atropine groups [13]. Similarly, Wei S et al. demonstrated axial length changes of 0.32±0.19 mm (0.01% atropine) versus 0.41±0.19 mm (placebo) [16].

Chia et al. also reported axial length elongations of 0.75±0.48 mm (0.01%), 0.85±0.53 mm (0.1%), and 0.87±0.49 mm (0.5%), indicating less axial elongation with lower doses [9]. Moon and Shin further corroborated that 0.05% atropine had the least axial elongation among the different concentrations studied [15].

Zhao et al., through a meta-analysis, verified that 0.05% atropine resulted in slower myopia progression and less axial elongation compared to higher concentrations [18].

In our study, the 0.05% atropine group (43.3%) had the highest proportion of children with the slowest progression (<0.25D/year), supporting its superior efficacy compared to 0.01% atropine and placebo. Conversely, the placebo group had the highest proportion (42.5%) of children with fast progression (≥1D/year).

Effects on accommodation and pupil size

Atropine treatment increased the near point of accommodation (NPA) and pupil size in both concentrations: (i) NPA increased by 18% in the 0.05% group and 10% in the 0.01% group; (ii) pupil size increased by 18% in the 0.05% group and 7.6% in the 0.01% group.

These findings align with previous reports by Chia et al. and Resnikoff et al., indicating that atropine affects ciliary muscle function and leads to accommodation difficulties and photophobia [9,19]. However, the side effects were generally mild and did not significantly impact compliance [9,19].

Moon and Shin found no statistically significant differences between the groups concerning accommodation issues or photophobia, supporting the relative safety of 0.05% atropine [15].

Zhao et al. also concluded that 0.05% atropine is the optimal dose, balancing efficacy and safety, with minimal rebound effects after cessation [18].

Our results confirm that while both concentrations of atropine are effective compared to placebo, 0.05% atropine provides superior control of myopia progression and axial elongation without significant adverse effects.

Safety and compliance

The safety profile observed in our study aligns with existing literature. Side effects, including mild photophobia and near vision difficulties, were manageable and did not lead to significant discontinuation. This supports the continued use of low-dose atropine as a safe and effective strategy for myopia management in children [20-21].

Limitations

We did not assess the association between parental myopia and axial length progression in children.

The follow-up duration was limited to one year, which, while sufficient to observe short-term trends, does not capture long-term effects or the rebound phenomenon after treatment cessation.

## Conclusions

This randomized interventional study demonstrated that both 0.01% and 0.05% atropine eye drops are effective in slowing myopia progression in Indian children compared to placebo, with 0.05% atropine showing superior efficacy. The 0.05% atropine group exhibited significantly less myopic progression and axial length elongation than the 0.01% group and placebo, aligning with findings from previous international studies. Additionally, while both concentrations caused mild increases in pupil size and near vision difficulties, these side effects were well-tolerated and did not significantly affect treatment compliance, supporting the safety of atropine at these low doses.

Our results emphasize that 0.05% atropine may be an optimal balance between efficacy and tolerability in controlling myopia progression. However, longer follow-up studies are needed to assess long-term safety, potential rebound effects after treatment cessation, and the influence of genetic and environmental factors. Despite these limitations, this study contributes valuable evidence supporting the use of low-concentration atropine in the Indian pediatric population, encouraging broader implementation of pharmacological strategies for myopia control.
